# PCB 153 Modulates Genes Involved in Proteasome and Neurodegeneration-Related Pathways in Differentiated SH-SY5Y Cells: A Transcriptomic Study

**DOI:** 10.3390/cells15030217

**Published:** 2026-01-23

**Authors:** Aurelio Minuti, Serena Silvestro, Claudia Muscarà, Michele Scuruchi, Simone D’Angiolini

**Affiliations:** 1 IRCCS Centro Neurolesi “Bonino-Pulejo”, Via Provinciale Palermo, Contrada Casazza, 98124 Messina, Italyclaudia.muscara@irccsme.it (C.M.);; 2 Department of Clinical and Experimental Medicine, University of Messina, 98124 Messina, Italy

**Keywords:** polychlorinated biphenyls, neurotoxicity mechanisms, environmental pollutants, PCB 153, exposure assessment, risk characterization, transcriptomic analysis

## Abstract

Polychlorinated biphenyls (PCBs) are persistent environmental contaminants associated with neurotoxicity and increased risk of neurodegenerative diseases. PCB 153, a highly abundant non-coplanar congener, bioaccumulates in human tissues and impairs homeostasis. This study investigated the transcriptomic effects of PCB 153 (2,2′,4,4′,5,5′-Hexachlorobiphenyl) in retinoic acid (RA)-differentiated SH-SY5Y neuronal cells to identify early, sub-cytotoxic molecular alterations. Cell viability was assessed by 3-(4,5-dimethylthiazol-2-yl)-2,5-diphenyltetrazolium bromide (MTT) assay after 24 h exposure to increasing PCB 153 concentrations. RNA-Seq was performed on cells treated with 5 μM PCB 153, the highest non-cytotoxic dose. Sequencing reads were quality-filtered, aligned to the human genome, and analyzed with DESeq2. Functional enrichment was conducted using Gene Ontologies and KEGG pathways. Western blot analyses were performed to assess protein level changes in selected targets. RNA-Seq identified 1882 significantly altered genes (q-value < 0.05). Gene Ontology analysis revealed strong enrichment of proteasome-related terms, with most proteasomal subunits displaying coordinated upregulation. KEGG analysis further showed significant enrichment of Alzheimer’s (AD), Parkinson’s (PD), amyotrophic lateral sclerosis (ALS), and other neurodegenerative disease pathways. These findings indicate that PCB 153 triggers a pronounced proteostatic response in neuron-like cells, suggesting early disruption of protein homeostasis that may contribute to mechanisms associated with neurodegeneration.

## 1. Introduction

**Figure 1:**
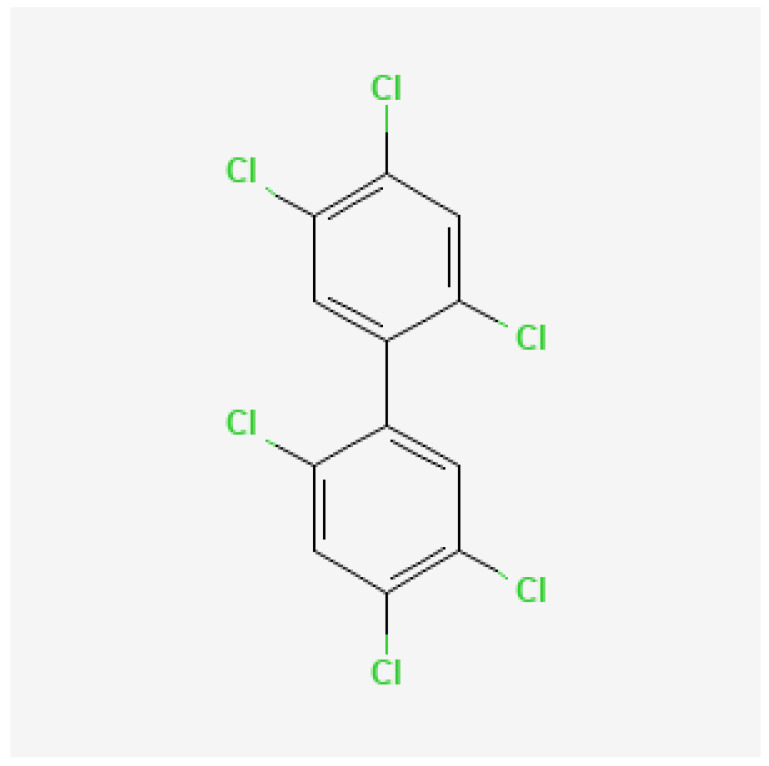
The chemical structure of the noncoplanar congener PCB 153 obtained from the PubChem Compound Summary [28]. Information regarding the molecular properties is provided at 2,2′,4,4′,5,5′-Hexachlorobiphenyl|C12H4Cl6|CID 37034–PubChem (https://pubchem.ncbi.nlm.nih.gov/compound/pcb-153 accessed on 17 October 2025).

Polychlorinated biphenyls (PCBs) are a family of 209 synthetic halogenated aromatic hydrocarbons consisting of one to ten chlorine atoms attached to a biphenyl structure [1]. PCB congeners are typically categorized according to the degree of ortho substitution: non-ortho congeners adopt a coplanar configuration, whereas mono- to tetra-ortho-substituted congeners exhibit progressively non-coplanar conformations (Figure 1) [2]. Although PCBs were banned in the United States in the late 1970s [3], they continue to be unintentionally generated and persist in consumer products such as paints, sealants, and polymeric materials [4,5,6]. Their chemical stability and lipophilicity promote bioaccumulation in adipose tissue and facilitate penetration across the blood–brain barrier [7].

Multiple studies have quantified PCB accumulation across tissues in both humans and experimental animals. For instance, in rats repeatedly exposed to PCB mixtures, total PCB concentrations were substantially higher in adipose tissue (~552 µg/g) than in blood (~38 µg/g), with measurable accumulation also being observed in brain regions [8]. In human fetal samples, median PCB levels (sum of key congeners) were reported at approximately 235 ng/g lipid in adipose tissue, 198 ng/g in liver, and 50 ng/g in brain [9].

Inhalation of lower-chlorinated congeners has recently emerged as a particularly relevant exposure route in indoor and occupational settings [10]. A substantial body of epidemiological and experimental evidence indicates that PCBs adversely affect the nervous system [11]. Neurobehavioral impairments, including deficits in memory, learning, and verbal skills have been reported in both children and adults exposed to PCBs [12]. Elevated PCB concentrations have also been associated with increased risk of neurodegenerative diseases, including Alzheimer’s disease (AD) [1], amyotrophic lateral sclerosis (ALS) [13], and Parkinson’s disease (PD) [14]. Occupational PCB exposure correlates with increased PD risk, particularly among women [15], and congeners such as PCB 153 and PCB 180 have been detected at higher levels in the caudate nucleus of PD patients compared with controls [16]. PCB 153 (2,2′,4,4′,5,5′-Hexachlorobiphenyl), a non-coplanar and highly chlorinated congener, is among the most persistent and abundant congeners in biological samples [17]. PCB 153 exposure has been linked to diverse adverse outcomes, including developmental toxicity [18], immune dysfunction [19], metabolic disorders like type II diabetes [20], thyroid dysregulation [21], and reproductive abnormalities [22]. Despite strong associations between PCB exposure and neurotoxicity, the molecular mechanisms underlying these effects remain insufficiently understood. These complexities are even more complicated by the difficulty of studying these mechanisms in people, since ethical and practical limitations restrict the use of controlled exposure experiments. SH-SY5Y, a human neuroblastoma cell line, is frequently used as an in vitro neuronal model [23,24]. When treated with retinoic acid (RA), these cells undergo differentiation characterized by neurite extension and increased expression of neuronal markers [25,26]. RA-differentiated SH-SY5Y cells are therefore considered a valuable surrogate model for studying neurodegenerative processes [27]. While several toxicological studies have investigated PCBs in neuronal systems; transcriptomic analyses of individual congeners, particularly PCB 153, in human neuron-like cells remain scarce. The present study fills the gap by performing a comprehensive RNA-Seq based analysis of RA-differentiated SH-SY5Y exposed to sub-cytotoxic PCB 153 concentrations. We identified a coordinated transcriptional response centered on proteasome components, revealing a previously uncharacterized mechanism by which PCB 153 may disrupt neuronal proteostasis. These findings provide new mechanistic insights into the neurotoxicity of a prevalent non-coplanar PCB congener.

## 2. Materials and Methods

### 2.1. Chemical Reagents

PCB 153 was obtained from LGC Standards (Milan, Italy). Dimethyl sulfoxide used for stock solutions and treatments was purchased from Sigma-Aldrich (Sigma-Aldrich, Saint Louis, MO, USA). All chemicals were sterile-filtered prior to use to prevent bacterial contamination.

### 2.2. Cell Culture and Treatment

The human SH-SY5Y neuroblastoma cell line was purchased from the American Type Culture Collection (ATCC; Manassas, VA, USA). Cells were plated either in 96-well plates (Corning Incorporated; Corning, NY, USA) at 4.0 × 10^4^ cells/well or in 6-well plates (ThermoFisher Scientific; Rochester, NY, USA) at 5.0 × 10^5^ cells/well. Cultures were grown in maintenance medium composed of DMEM/F-12 Ham (Sigma-Aldrich; St. Louis, MO, USA) supplemented with 10% fetal bovine serum, 1% penicillin/streptomycin, and 1% L-glutamine (Sigma-Aldrich; St. Louis, MO, USA), and maintained at 37 °C in a humidified incubator with 5% CO_2_. Neuronal differentiation was induced the day after seeding by treating cells with 10 μM retinoic acid (RA) (Sigma-Aldrich; St. Louis, MO, USA) for 5 days. After differentiation, cells were exposed to different concentrations of PCB 153 for 24 h.

### 2.3. MTT Assay

Differentiated SH-SY5Y cells were plated in 96-well plates and exposed to PCB 153 as described above. At the end of the exposure, cell viability was evaluated by the 3-(4,5-dimethylthiazol-2-yl)-2,5-diphenyltetrazolium bromide (MTT) assay (Sigma-Aldrich, St. Louis, MO, USA). In brief, cells were incubated with maintenance medium supplemented with 0.5 mg/mL MTT for 4 h at 37 °C. Formazan crystals were then solubilized in acidified isopropanol (0.1 N HCl) for 1 h at 37 °C, and absorbance was measured at 570 nm using a BioTek Synergy H1 microplate reader (Agilent, Santa Clara, CA, USA).

### 2.4. RNA Extraction and cDNA Library Preparation

SH-SY5Y cells were seeded in 6-well plates and exposed to the indicated treatments. Following treatment, cells were harvested by detachment with 0.25% trypsin–EDTA (Sigma-Aldrich, St. Louis, MO, USA) and centrifuged at 300× *g* for 5 min to collect the cell pellets. Total RNA was then extracted using the Maxwell^®^ RSC simplyRNA Cells Kit (Promega, Madison, WI, USA) on the Maxwell^®^ RSC Instrument, following the manufacturer’s protocol. Library preparation was performed using 100 ng of total RNA (two biological replicates) using the TruSeq^®^ RNA Exome Kit (Illumina, San Diego, CA, USA) according to the manufacturer’s instructions. Library quality was assessed using the TapeStation 4150 system (Agilent, Santa Clara, CA, USA) with D1000 ScreenTape reagents (Agilent, Santa Clara, CA, USA). Prior to sequencing, libraries were denatured using 0.2 N NaOH and then diluted to a final concentration of 1.42 pM. Sequencing was carried out on an Illumina NextSeq™ 550Dx instrument (Illumina, San Diego, CA, USA) using the NextSeq 500/550 Mid Output Reagent Kit v2.5 (150 cycles) in paired-end mode.

### 2.5. Transcriptomic Analysis

To ascertain the quality and fidelity of the sequencing data utilized in this investigation, an initial quality check of the raw reads was executed employing FastQC v.0.12.0 (Babraham Institute, Cambridge, UK) (FastQC. A Quality Control Tool for High Throughput Sequence Data. Available online: https://qubeshub.org/resources/fastqc, accessed on 20 October 2025). The sequence reads were then subjected to rigorous quality trimming and adapter removal using Trimmomatic v.0.40-rc1 (Usadel Lab, Aachen, Germany) [29], which eliminated low-quality bases and contaminating adapter sequences. The resulting high-quality reads were subsequently aligned to the GENCODE hg38 v39 reference genome using the STAR RNA-seq aligner 2.7.10a_alpha_220207 (New York, NY, USA) [30]. Following successful mapping, transcript abundance per gene was accurately quantified using HTSeq v.0.13.5 [31]. Differential Gene Expression analysis was performed using the DESeq2 package v.1.36.0 [32] within R statistical environment v.4.2.0 (R Core Team). This methodology utilizes generalized linear models based on the negative binomial distribution, which is highly suitable for count data derived from RNA sequencing. To rigorously control the False Discovery Rate (FDR), the derived *p*-values were adjusted via the Benjamini–Hochberg correction, with Differentially Expressed Genes (DEGs) being defined by a stringent threshold of q-value < 0.05. List of DEGs was then assessed for functional significance via Gene Ontology (GO) over-representation analysis (ORA) utilizing the clusterProfiler R package v4.6.2 [33]. ORA *p*-values were corrected using the same step of DEGs analysis maintaining all the ontologies with a q-value < 0.05. Complementary understanding of the cellular pathways impacted by the DEGs was gained by consulting the Kyoto Encyclopedia of Genes and Genomes (KEGG) database [34].

### 2.6. Protein Extraction and Western Blot Analysis

SH-SY5Y cells were harvested using trypsin-EDTA following treatment. Protein extraction was performed with NE-PER Nuclear and Cytoplasmic Extraction kit (Thermo Scientific™, Waltham, MA, USA) according to the manufacturer’s protocol. Protein concentrations were determined using the Bradford assay (Bio-Rad, Hercules, CA, USA). Equal amounts of protein (20 µg per sample) were resolved by sodium dodecyl sulfate-polyacrylamide gel electrophoresis (SDS-PAGE) and subsequently transferred to Polyvinylidene fluoride (PVDF) membranes (Immobilon-P, Merck Millipore, Darmstadt, Germany). The membranes were then blocked with 5% skim milk prepared in TBS for 1 h at room temperature and incubated overnight at 4 °C with the appropriate primary antibodies. The following primary antibodies were utilized: anti-Bax (1:1000, Cell Signaling, Danvers, MA, USA), S6 Ribosomal Protein (1:1000, Cell Signaling, Danvers, MA, USA), and anti-IKB-α (1:1000, Sigma-Aldrich, St. Louis, MO, USA). Glyceraldehyde 3 phosphate dehydrogenase (GAPDH) was used as a loading control using an anti-GAPDH HRP-conjugated antibody (1:1000, Cell Signaling, Danvers, MA, USA). A mouse anti-rabbit IgG–HRP secondary antibody (1:2000; Santa Cruz Biotechnology, Dallas, TX, USA) and a chicken anti-Mouse IgG (1:2000 ThermoScientific™, Waltham, MA, USA) were applied to the membranes for 1 h at room temperature. Immunoreactive bands were detected using an enhanced chemiluminescence reagent (Luminata Western HRP Substrates; Millipore Corporation, Billerica, MA, USA). Signals were acquired and densitometrically quantified with a ChemiDoc™ MP Imaging System (Bio-Rad Laboratories S.r.l., Hercules, CA, USA), and band intensities were analyzed using ImageJ (v1.54j).

### 2.7. Statistical Analysis

Statistical analyses were performed using GraphPad Prism software, version 10.1 (GraphPad Software, La Jolla, CA, USA). Differences among groups were evaluated by one-way ANOVA followed by Bonferroni’s post hoc test for multiple comparisons. A *p*-value ≤ 0.05 was considered indicative of statistical significance. All data are presented as the mean ± standard deviation (SD).

## 3. Results

### 3.1. Effects of PCB 153 on Cell Viability

**Figure 2:**
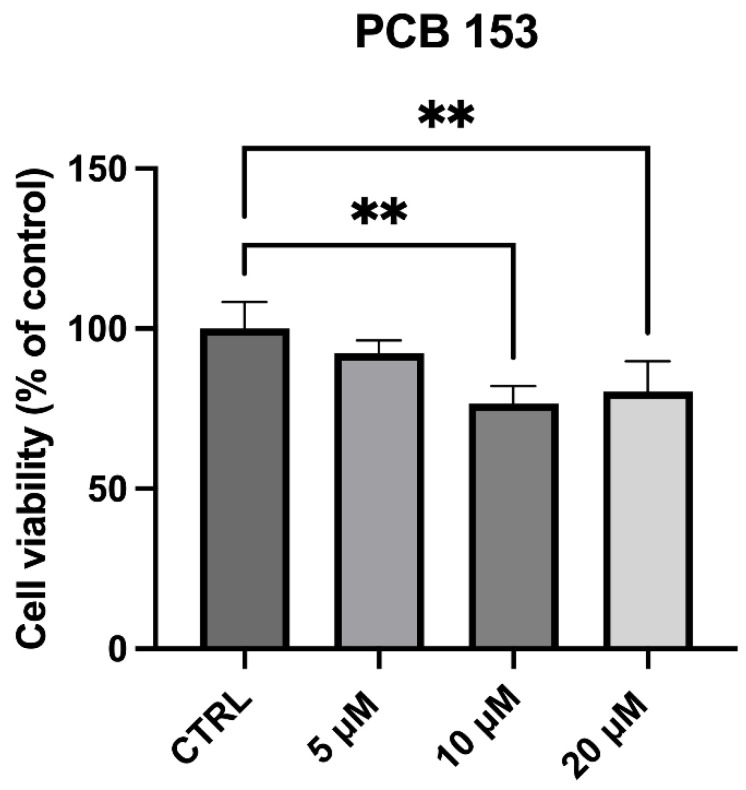
Effect of PCB exposure on cell viability of differentiated SH-SY5Y cells with increasing concentrations (5, 10, and 20 µM) of PCB 153. Cell viability was assessed after 24 h of exposure. Data are expressed as the mean ± SD of eight independent experiments. ** *p* < 0.001 vs. CTRL.

Viability assays performed on differentiated SH-SY5Y cells exposed to increasing concentrations (5, 10, and 20 µM) of PCB 153 (Figure 2) revealed that higher doses reduced cell viability, whereas 5 µM concentration did not produce significant cytotoxic effects. As viability was not significantly altered at 5 µM, this concentration was chosen for the subsequent transcriptomic analysis to ensure that observed gene expression changes were not secondary to cytotoxic effects.

### 3.2. Differential Expression Analysis

**Figure 3:**
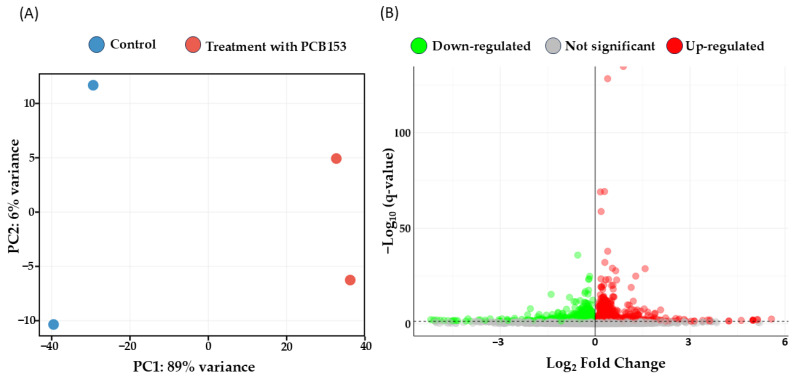
Global transcriptomic assessment of PCB 153 impact. (**A**) Principal Component Analysis (PCA) performed on normalized counts, illustrating the clustering of biological replicates, 2 for each condition. The first principal component (PC1), accounting for 89% of the total variance, shows the separation between Control (blue) and PCB 153-treated (red) SH-SY5Y cells. (**B**) Volcano plot displaying all transcripts inspected in the comparison. The x-axis represents the Log_2_ Fold Change (FC), while the y-axis indicates the statistical significance expressed as −Log_10_ q-value. The horizontal dashed line marks the significance threshold (q-value < 0.05).

To evaluate the transcriptomic impact of PCB 153, a Principal Component Analysis (PCA) was performed on the normalized counts to assess sample clustering and the primary sources of experimental variance. As illustrated in Figure 3A, the PCA shows a distinct separation between experimental groups along the first principal component (PC1), which accounts for 89% of the total variance. This distribution suggests that the PCB 153 treatment represents a major factor contributing to the observed molecular changes, with biological replicates showing consistent clustering within their respective conditions. Subsequently, a differential gene expression analysis was conducted on RA-differentiated SH-SY5Y cells treated with 5 µM PCB 153 for 24 h. To control the false discovery rate (FDR) from multiple testing, *p*-values were adjusted using the Benjamini–Hochberg method. DEGs were defined using a threshold of q-value < 0.05, without an a priori fold-change (FC) cutoff, to ensure a comprehensive capture of statistically robust biological alterations. This approach identified 1882 DEGs, comprising 1048 up-regulated and 834 down-regulated transcripts. The full list of transcripts with their associated *p* and q-values is provided in Supplementary Table S1. The magnitude and statistical significance (−log_10_ q-value) of these alterations are visualized in the Volcano Plot (Figure 3B).

### 3.3. Over-Representation Analysis

**Figure 4:**
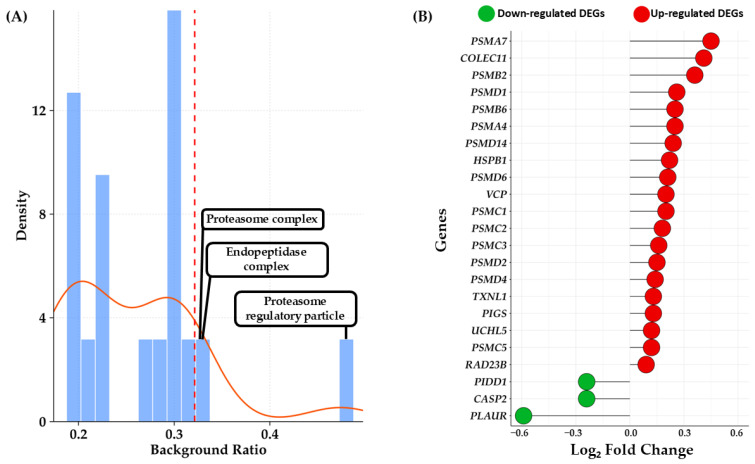
In panel (**A**) is reported the histogram illustrates the density distribution of the background ratio for the significantly enriched GO. The red dashed line highlights the 90th percentile while the solid one illustrates the distribution shape of the density. Panel (**B**) shows Lollipop plot detail about Log_2_ Fold Change values for the 23 DEGS identified as contributing to the enrichment of the proteasome system.

To interpret the biological significance of the 1882 DEGs identified in the cells treated with PCB 153, we conducted an ORA. The ORA serves as a foundational approach that aims to determine whether a known set of genes is observed more often than expected by chance within predefined biological pathways or GO terms. This analysis helps translate long lists of expression changes into manageable, biologically meaningful concepts. The analysis was performed using all the DEGs resulted from the analysis, both up and down-regulated, through the clusterProfiler R package, a widely recognized tool for functional enrichment analysis. The ORA specifically tested for the enrichment of GO terms across three major categories: Biological Process (BP), Cellular Component (CC), and Molecular Function (MF). For the statistical definition of enrichment, we maintained a strict threshold. The *p*-values derived from the statistical tests were adjusted using the Benjamini–Hochberg (BH) correction method. This crucial step controls the FDR, which is the expected proportion of false positives among all rejected null hypotheses. Setting a stringent threshold of q-value < 0.05, we ensured that the identified ontologies were statistically robust, mitigating the high risk of false discoveries inherent in analyzing thousands of genes simultaneously. The results of the ORA are fully available in Supplementary Table S2 and here resumed in Figure 4A. Crucially, when ranking all significant GO terms by their background ratio, the top three absolute terms converged exclusively on the cellular machinery responsible for protein quality control and degradation. These three most highly enriched ontologies were all classified under the CC category: GO:0005838 (Proteasome Regulatory Particle), GO:1905369 (Endopeptidase Complex), GO:0000502 (Proteasome Complex). The strong and singular focus of the top results on the Proteasome System emphasizes the transcriptional vulnerability of proteostasis in response to PCB 153. Based on these top enriched terms, the specific set of genes contributing to the enrichment of the entire proteasome system was extracted. These genes were then compiled into a unique, non-redundant list, resulting in 23 DEGs. The transcriptional impact on this key pathway was subsequently assessed by examining the Log_2_ FC values for every gene in this curated list, represented in the lollipop plot shown in Figure 4B.

**Figure 5:**
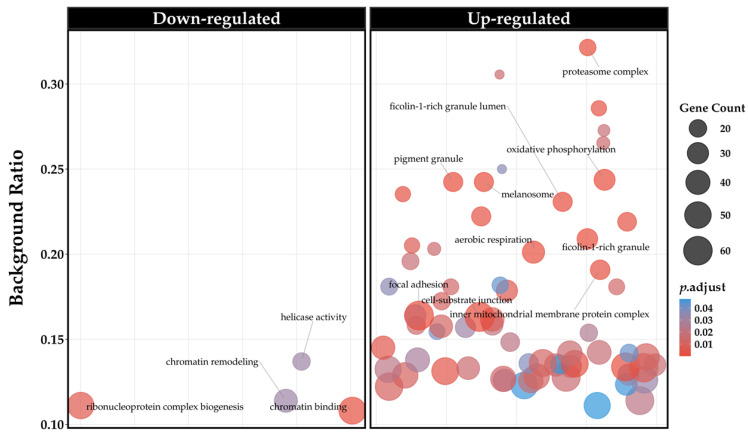
Global overview of GO enrichment analysis. Cloud Bubble Plot displaying significantly enriched GO terms for up-regulated (**left**) and down-regulated (**right**) genes. The *y*-axis represents the Relative Enrichment Score; bubble size indicates gene count, and color indicates the adjusted *p*-value.

As detailed in Figure 4B, the majority of the identified genes showed an upregulation in expression following PCB 153 treatment. Specifically, 20 of the 23 DEGs exhibited positive Log_2_ FC values, indicating increased expression, while only 3 genes (*CASP2*, *PIDD1*, and *PLAUR*) were downregulated. To better characterize the directional impact of PCB 153 and address the potential masking of pathways, we performed two separate ORA analyses for up-regulated and down-regulated genes. This approach revealed a striking asymmetry in the functional response: 67 biological processes were significantly up-regulated, whereas only 4 were down-regulated. The global distribution of these enriched terms is summarized in the Cloud Bubble Plot (Figure 5), where the density of terms visually emphasizes the massive transcriptional activation compared to the localized repression. The full list of these directional ontologies is provided in Supplementary Table S3. Notably, even when analyzed separately, the top enriched terms remained consistently linked to protein degradation.

The consistent upregulation of core proteasome components underscores a strong and coordinated transcriptional response, likely reflecting a compensatory mechanism aimed at enhancing protein quality control (proteostasis) in response to PCB 153-induced stress. Using this approach, it was possible to observe additionally element related to proteostasis also in down-regulated ontologies considering the down-regulation of the complex related to ribonucleoprotein biogenesis as reported in Figure 5.

### 3.4. Network and Pathways Analysis

**Figure 6:**
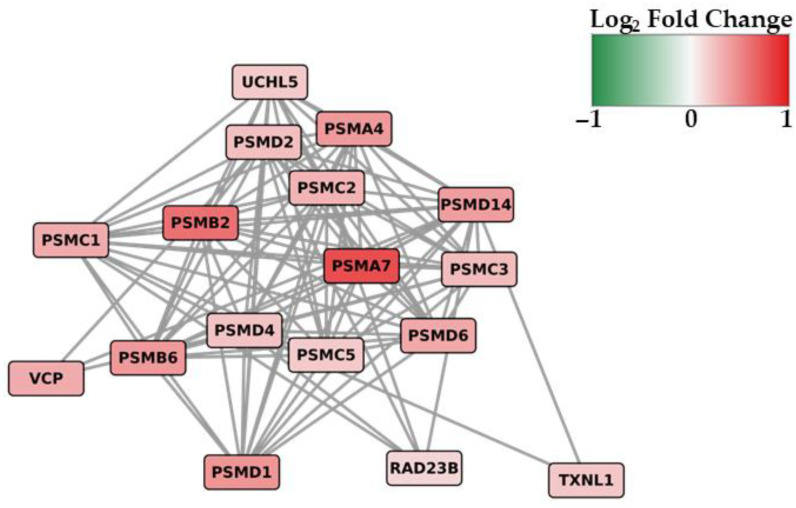
Protein–Protein Interaction (PPI) network analysis of the DEGs related to the proteasome system. The color intensity of each node represents the Log2 Fold Change (FC) value, with red indicating up-regulation and green indicating down-regulation.

To gain a deeper understanding of the functional relationships and interconnectivity among the proteins encoded by the 23 proteostasis-associated DEGs highlighted in Figure 4B, a protein–protein interaction (PPI) network analysis was performed. The resulting network, shown in Figure 6, specifically highlights the 17 proteins that exhibited documented interactions, forming a cohesive functional cluster. The remaining 6 DEGs were excluded from the visualization as they appeared as isolated nodes without known interactions within this specific set.

The PPI network shown in Figure 6 visually reinforces the findings from the Lollipop Plot (Figure 5). As evidenced by the red-colored nodes, the core and regulatory subunits of the proteasome system exhibit an upregulation. To gain a more comprehensive understanding of the broader cellular processes perturbed by the 1882 DEGs, we subsequently performed a KEGG pathway enrichment analysis. This approach is important for the identification of potential molecular mechanisms and disease associations beyond the singular focus on the proteasome structure initially highlighted by the GO analysis. The KEGG analysis results, which highlight the significantly enriched pathways, are presented in Table 1.

**Figure 7:**
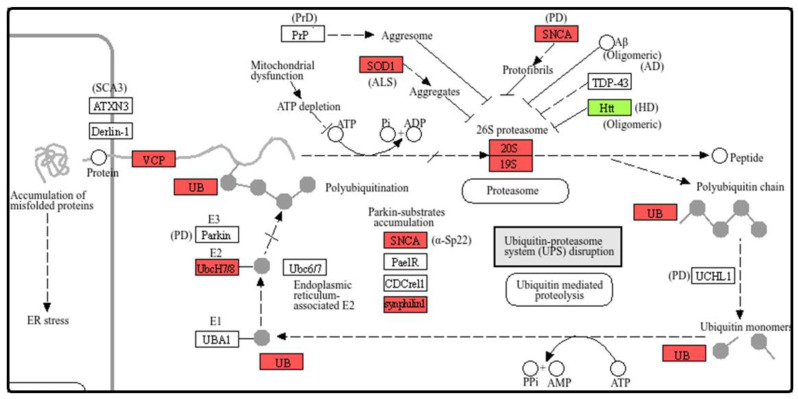
In the pathways the red boxes indicate upregulated DEGs while the green boxes indicate downregulated DEGs.

The KEGG enrichment analysis concurred with the GO results by identifying the Proteasome (hsa03050) as a significantly impacted pathway, but also revealed a pronounced transcriptional impact on pathways associated with several major neurodegenerative diseases, including AD, PD, ALS, Huntington disease, and prion disease. Given this consistent pattern, we specifically focused on the general “Pathways of neurodegeneration–multiple diseases” (hsa05022) pathway, which integrates many of the individual neurodegenerative diseases reported in the list. To visualize the specific transcriptional changes within this context, we extracted the portion of the neurodegeneration pathway directly linked to the ubiquitin-proteasome system (UPS). The complete pathway image is available in the Supplementary Figure S1, but in the following Figure 7 is reported the portion relevant to the proteasome and its related components within the neurodegeneration cascade. Figure 7 illustrates the specific genes from the enriched “Pathways of neurodegeneration–multiple diseases” that were differentially expressed in our PCB 153 treated cells.

### 3.5. Western Blot Analyses

**Figure 8:**
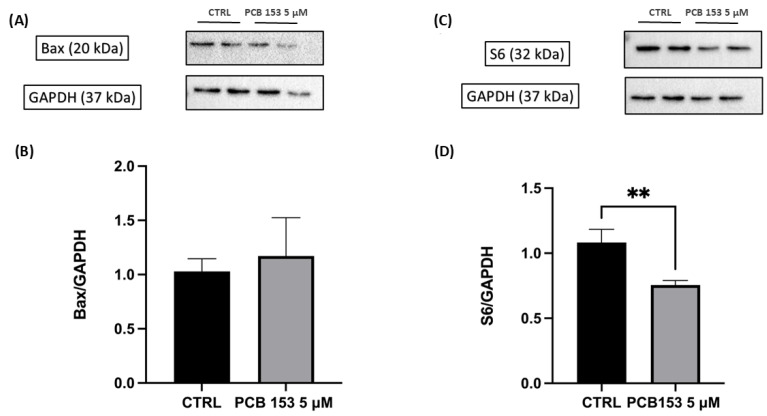
Effects of PCB 153 on BAX and ribosomal protein S6 in RA-differentiated SH-SY5Y cells. (**A**) Representative Western blots of BAX and GAPDH in control (CTRL) and PCB 153 (5 µM)–treated cells. (**B**) Densitometric analysis of BAX expression normalized to GAPDH (BAX/GAPDH), showing no significant difference between CTRL and PCB 153. (**C**) Representative Western blots of S6 and GAPDH in CTRL and PCB 153–treated cells. (**D**) Densitometric analysis of S6 expression normalized to GAPDH (S6/GAPDH), indicating a significant reduction in S6 levels after PCB 153 exposure. Data are expressed as mean ± SD of at least two independent experiments; ** *p*-value < 0.01 vs. CTRL.

Western blot analysis showed that Bax protein levels were not significantly affected by exposure to PCB 153 (Figure 8A,B), indicating the absence of mitochondrial pro-apoptotic activation under these sub-cytotoxic conditions. In contrast, a significant decrease in S6 protein levels was detected in PCB-treated cells (Figure 8C,D). As S6 is a key component of the 40S ribosomal subunit involved in translational control, its downregulation suggests an early alteration of the neuronal protein synthesis machinery following PCB 153 exposure. Data are presented as mean ± SD. Asterisk (**) indicate *p*-value: ** *p* < 0.01.

**Figure 9:**
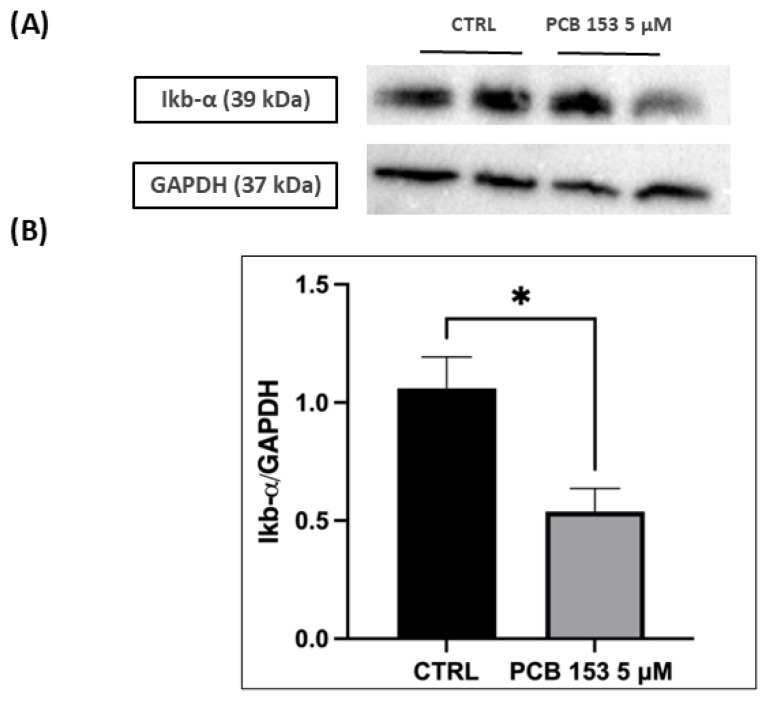
Effects of PCB 153 on Ikb-α in RA-differentiated SH-SY5Y cells. (**A**) Representative Western blots of Ikb-α and GAPDH in control (CTRL) and PCB 153 (5 µM)–treated cells. (**B**) Densitometric analysis of Ikb-α expression normalized to GAPDH (Ikb-α/GAPDH), showing significant difference between CTRL and PCB 153. Data are expressed as mean ± SD of at least two independent experiments; * *p*-value < 0.05 vs. CTRL.

Moreover, as shown in Figure 9, Western blot analysis revealed a significant decrease in IκB-α protein levels in cells treated with PCB 153 (5 µM) compared to control cells. Representative immunoblots show a reduced IκB-α signal as confirmed by densitometric quantification normalized to GAPDH (*p* < 0.05 (Figure 9).

## 4. Discussion

A growing body of evidence suggests that environmental pollutants, including persistent organic contaminants such as PCBs, may contribute to neuronal vulnerability and influence the onset or progression of neurodegenerative diseases [35]. In this context, investigating how specific pollutants such as PCB 153 affect neuronal cells, even at sub-cytotoxic concentrations, is relevant for understanding transcriptomic responses to environmental factors.

In this study, we examined the transcriptional effects of PCB 153 in RA-differentiated SH-SY5Y cells, a well-established neuronal model widely used in neurotoxicity and neurodegeneration research [25]. Consistent with our MTT results, 5 µM PCB 153 did not alter cell viability, and was therefore selected for transcriptomic analysis, allowing for the assessment of early or subtle gene expression changes independent of overt cytotoxicity.

The differential expression analysis revealed a large set of transcriptional alterations, with 1882 DEGs, including 1048 upregulated and 834 downregulated genes. This highlights that differentiated neuronal cells exhibit pronounced transcriptional responses to PCB 153 exposure.

Interestingly, a key and novel outcome of our ORA is the strong enrichment of GO categories associated with the proteasome. The top three most enriched GO terms were: Proteasome Regulatory Particle (GO:0005838), Endopeptidase Complex (GO:1905369), and Proteasome Complex (GO:0000502), all point to a coordinated activation of the protein degradation machinery. The proteasome is a highly dynamic intracellular system responsible for degrading damaged or misfolded proteins and regulating overall protein turnover. It is made up of a 20S core particle flanked by one or two regulatory particles 19S at one or both ends. The 20S proteasome consists of 28 protein subunits organized into four stacked rings, each containing seven subunits, forming an α7β7β7α7 structure [36]. During its assembly into the ubiquitin-dependent degradation machinery, the 20S proteasome associates with one or two 19S regulatory particles to form the 26S proteasome [37]. As the central catalytic component of the UPS, it plays a crucial role in controlling protein homeostasis and sustaining proper cellular signaling [38]. In the nervous system, the proteasome plays a key role in protein degradation and in preserving cellular homeostasis across neurons and glial cells, thereby contributing to overall brain function. Increasing evidence also indicates that the proteasome fulfills specialized role in neurons, particularly in processes underlying long-term facilitation [39], potentiation [40], and neurodevelopment [41].

Impairments in the mechanisms that maintain protein balance and mediate protein degradation are closely linked to various neurodegenerative diseases [42]. This result is particularly striking because it suggests that PCB 153 exposure may compromise neuronal proteostasis, triggering compensatory upregulation of proteasomal components. Indeed, 20 out of 23 proteasome-related DEGs (*PSMA7*, *COLEC11*, *PSMB2*, *PSMD1*, *PSMA4*, *PSMB6*, *PSMD14*, *HSPB1*, *PSMD6*, *PSMC1*, *VCP*, *PSMC2*, *PSMC3*, *PSMD2*, *PSMD4*, *PIGS, TXNL1*, *PSMC5*, *UCHL5*, and *RAD23B*) showed positive FC values, indicating enhanced transcription of catalytic and regulatory subunits involved in ubiquitin-dependent degradation. Notably, exposure to 5 μM PCB 153 resulted in the upregulation of *PSMA4*, *PSMA7*, *PSMB2*, and *PSMB6* that belong to the 20S proteasomal core, and also increased 26S subunit genes *PSMD1*, *PSMD2*, *PSMD4*, *PSMD6*, *PSMD14*, *PSMC1*, *PSMC2*, *PSMC3*, *PSMC4*, and *PSMC5*. Additional UPS-related genes (*UCHL5*, *RAD23B*, *VCP*, and *HSPB1*) also showed increased transcript levels, consistent with coordinated transcriptional modulation of proteasome-related components. Interestingly, *RAD23B*, *VCP*, and *HSPB1* are involved not only in protein degradation but also in DNA repair and neuropathies [43,44,45]. Proteasomal activation is commonly observed in conditions involving protein misfolding, oxidative stress, or the accumulation of neurotoxic aggregates [46,47]. Notably, as shown in Figure 5, exposure to PCB 153 5 µM caused a downregulation of genes involved in ribonucleoprotein complex biogenesis was observed concomitantly with the upregulation of proteasome-related pathways. This transcriptional pattern suggests a coordinated cellular response in which reduced RNA processing and ribosome biogenesis may limit protein synthesis, while enhanced proteasomal activity may promote protein turnover and quality control under PCB 153-induced stress conditions.

Given that PCBs can generate reactive oxygen species [48], disrupt mitochondrial function [49], and destabilize neuronal membranes [50], the observed transcriptional changes may represent an early cellular response to PCB-induced stress at the transcriptomic level. These transcriptional changes are relevant in the context of neurodegeneration. This finding may be of high relevance for neurodegeneration research, as proteostasis collapse is a hallmark of disorders such as PD, AD, and Huntington’s disease [47]. PCB 153 exposure, even at nonlethal doses, is associated with modulation of proteasome-related genes, which may have implications for cellular processes linked to neurodegenerative susceptibility. Although the proteasome-related genes were predominantly upregulated, three DEGs (*CASP2*, *PIDD1*, and *PLAUR*) displayed decreased expression. *CASP2* and *PIDD1* are central components of the PIDDosome, a protein complex involved in apoptosis and DNA-damage sensing [51]. Their downregulation may reflect a compensatory attempt to repress apoptotic signaling during early PCB-induced stress. Similarly, reduced *PLAUR* expression may relate to altered extracellular remodeling or impaired neuroinflammatory communication. These findings suggest that PCB 153 elicits a multifaceted response balancing pro-survival and pro-apoptotic cues.

To further substantiate the involvement of stress- and apoptosis-related pathways suggested by our transcriptomic data, we also evaluated BAX protein levels by Western blot. In line with the RNA-seq results, PCB 153 exposure produced a trend toward increased BAX protein expression in RA-differentiated SH-SY5Y cells compared with vehicle-treated controls; however, this change did not reach statistical significance. BAX is a key effector of the intrinsic, mitochondria-dependent apoptotic pathway, and its upregulation is widely regarded as a marker of mitochondrial stress and pro-apoptotic priming [52]. The directional concordance between BAX transcript and protein changes therefore provides supportive, although not definitive, validation of our omics findings at the protein level and suggests that, even at non-cytotoxic concentrations, PCB 153 may begin to shift intracellular signaling toward a state of heightened vulnerability, in which neurons remain viable but are closer to the apoptotic threshold. When considered together with the downregulation of CASP2 and PIDD1, these data further reinforce the notion that PCB 153 elicits a complex adaptive response that simultaneously enhances apoptotic readiness while engaging compensatory mechanisms to delay full execution of cell death.

Furthermore, in line with the transcriptomic evidence indicating an upregulation of the proteasomal machinery, we observed a significant downregulation of IκB-α protein levels, as assessed by Western blot analysis. IκB-α is a well-established substrate of ubiquitin-dependent proteasomal degradation, and is the canonical inhibitory protein of NF-κB, retaining NF-κB in the cytoplasm and preventing its nuclear translocation and transcriptional activity. Alterations in proteasomal activity directly impact IκB-α stability and NF-κB signaling [53].

KEGG pathway enrichment further reinforced these findings. In addition to confirming the proteasome pathway (hsa03050) as significantly affected, the analysis revealed marked enrichment of several neurodegeneration-related pathways, including those for AD, PD, Huntington’s disease, ALS, and prion disease. Notably, the “Pathways of Neurodegeneration–Multiple Diseases” pathway was also significantly enriched. These disease-specific pathways share key mechanistic nodes, such as mitochondrial impairment, oxidative stress, and proteasome dysfunction, that collectively reflect core processes underlying neurodegeneration. Neurodegeneration-related KEGG pathways are tightly interconnected with the proteasome-mediated protein degradation pathway, which is critical because the accumulation of misfolded proteins is a central hallmark of major neurodegenerative conditions [54]. The convergence of these pathways with the strong proteasome signature observed in our GO and DEG analyses suggests that PCB 153 disrupts molecular networks fundamental to neuronal proteostasis.

The PPI network of the 23 proteasome-related DEGs further highlights the functional coherence of the proteostasis machinery affected by PCB 153. The dense interconnections among core subunits indicate that the transcriptional response is not random but reflects a coordinated reprogramming of proteasome architecture. Upregulation of genes encoding both 19S regulatory and 20S catalytic proteasome components suggests a coordinated transcriptional modulation of proteasome-related pathway, potentially indicative of an altered transcriptional response to xenobiotic exposure [37,55].

From a broader perspective, these findings have important implications for environmental neurotoxicology. Proteostasis is essential for neuronal survival, and proteasome impairment or overload is strongly implicated in AD, PD, ALS, and Huntington’s disease [47]. Our findings indicate that PCB 153 triggers a strong proteostatic response in differentiated neuronal cells, even in the absence of cytotoxicity. This response may represent an early cellular attempt to maintain homeostasis in the face of PCB-induced disturbances in protein integrity, oxidative balance, or lipid signaling.

Furthermore, Western blot analysis revealed that PCB 153 (5 µM) significantly reduced the expression of the ribosomal protein S6. In line with the transcriptomic signature pointing to an involvement of protein synthesis and proteostasis-related pathways, we examined total S6 levels because this protein is both a core component of the 40S ribosomal subunit and a key integrator of environmental cues into translational control in neurons [56]. The observed decrease in S6 protein abundance in RA-differentiated SH-SY5Y cells exposed to PCB 153 compared with vehicle-treated controls is consistent with transcriptional and proteomic changes in pathways related to protein synthesis and proteostasis. S6 participates in the mTOR–S6K axis, regulating translation of mRNAs relevant to synaptic function; thus, reduced S6 expression may be associated with modulation of protein synthesis at synapses, without implying direct functional impairment [55]. Together with the proteasome-related and neurodegeneration-associated pathways highlighted by KEGG and GO analyses, the modulation of S6 supports the view that PCB 153 reshapes the balance between protein synthesis and degradation as part of an integrated adaptive response, potentially contributing to an altered proteostatic setpoint and increased neuronal vulnerability over time.

Given that neurons rely heavily on efficient proteostasis for long-term survival, especially in post-mitotic systems, chronic or repeated PCB exposure could, over time, saturate or compromise proteasome efficiency. This raises important questions regarding environmental PCB exposure as a potential modulator of neurodegenerative disease risk, particularly in individuals with pre-existing disruptions in proteostasis, mitochondrial function, or antioxidant defense.

**Figure 10:**
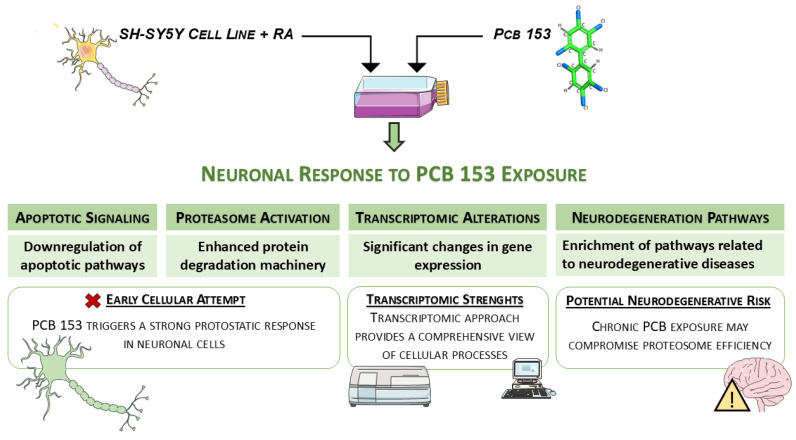
Schematic overview of the neuronal response to PCB 153 exposure in SH-SY5Y cells differentiated with RA. Exposure to PCB 153 induces marked transcriptomic alterations, activation of the proteasome, and modulation of apoptotic pathways, triggering an early pro-survival response but also enriching pathways linked to neurodegenerative diseases, thereby suggesting a potential neurodegenerative risk upon chronic exposure. The image was created using the image bank of Servier Medical Art (Available online: http://smart.servier.com/, accessed on 10 December 2025), licensed under a Creative Commons Attribution 4.0 (CC BY 4.0) (Available online: https://creativecommons.org/licenses/by/4.0/, accessed on 10 December 2025).

In conclusion, the strengths of this study lie primarily in the transcriptomic approach, which enabled the investigation of a broad spectrum of genes spanning multiple biological pathways. This methodology allows for the simultaneous assessment of numerous molecular targets, providing a more comprehensive and integrated view of complex cellular processes. Furthermore, the rigorous filtering strategy applied to our dataset minimizes false positives and facilitates the identification of the key pathways and core mechanisms specifically affected by PCB 153 (Figure 10).

Nonetheless, the exploratory nature of this study represents a limitation. Although we successfully identified several pathways of interest, targeted follow-up experiments will be required to validate and further characterize these findings. For example, dose-dependent effects may differ between in vitro and in vivo systems. Additional validation in primary neurons or in vivo systems would strengthen the biological relevance of our findings.

Future studies should explore whether proteasome modulation is a common response to other noncoplanar PCB congeners and assess the long-term consequences of proteasome activation or overload in neuronal health.

## Figures and Tables

**Table 1:** Significantly enriched KEGG pathways for the 1882 differentially expressed genes (DEGs).

Pathway ID	Description	Gene Ratio	q-Value
hsa03050	Proteasome	15/42	3.7 × 10^−2^
hsa05010	AD	75/315	1.4 × 10^−3^
hsa05012	PD	60/224	5.9 × 10^−4^
hsa05014	ALS	74/312	1.5 × 10^−3^
hsa05016	Huntington disease	65/260	1.2 × 10^−3^
hsa05020	Prion disease	58/215	5.9 × 10^−4^
hsa05022	Pathways of neurodegeneration–multiple diseases	91/397	1.2 × 10^−3^

AD: Alzheimer’s disease; PD: Parkinson’s disease; ALS: Amyotrophic lateral sclerosis.

## Data Availability

The data presented in this study are openly available in the NCBI Sequence Read Archive at BioProject; accession number: PRJNA1378135.

## Supplementary Materials

The following supporting information can be downloaded at https://www.mdpi.com/article/10.3390/cells15030217/s1. Figure S1: Complete pathways of neurodegeneration – multiple diseases, Figure S2: Full-length Western Blot membranes for Bax and GAPDH, Figure S3: Full-length Western Blot membranes for S6 and GAPDH, Figure S4: Full-length Western Blot membranes for Ikb-α and GAPDH, Table S1: Complete list of gene whit associated information, Table S2: Complete list of gene ontologies ORA, Table S3: Complete list of gene ontology ORA separated based on the FC

## Abbreviations

The following abbreviations are used in this manuscript:

AD	Alzheimer’s disease
ALS	Amyotrophic lateral sclerosis
ATCC	American Type Culture Collection
BP	Biological Process
CC	Cellular Component
DEGs	Differentially expressed genes
EDTA	Ethylenediaminetetraacetic acid
FC	Fold change
FDR	False discovery rate
GAPDH	Glyceraldehyde 3 phosphate dehydrogenase
GO	Gene Ontology
HRP	Horseradish peroxidase
KEGG	Kyoto Encyclopedia of Genes and Genomes
MTT	3-(4,5-dimethylthiazol-2-yl)-2,5-diphenyltetrazolium bromide
NaOH	Sodium hydroxide
ORA	Over-representation analysis
PCB	Polychlorinated biphenyl
PCB 153	2,2′,4,4′,5,5′-Hexachlorobiphenyl
PD	Parkinson’s disease
PPI	Protein–protein interaction
PVDF	Polyvinylidene fluoride
RA	Retinoic acid
SD	Standard deviation
SDS-PAGE	Sodium dodecyl sulfate polyacrylamide gel electrophoresis
TBS	Tris-buffered saline
UPS	Ubiquitin-proteasome system
