# Supplementary figures and images for: PCB 153 Modulates Genes Involved in Proteasome and Neurodegeneration-Related Pathways in Differentiated SH-SY5Y Cells: A Transcriptomic Study
Source: Cells. 2026 Jan 23;15(3):217. doi: 10.3390/cells15030217 (PMC12896660; doi:10.3390/cells15030217)

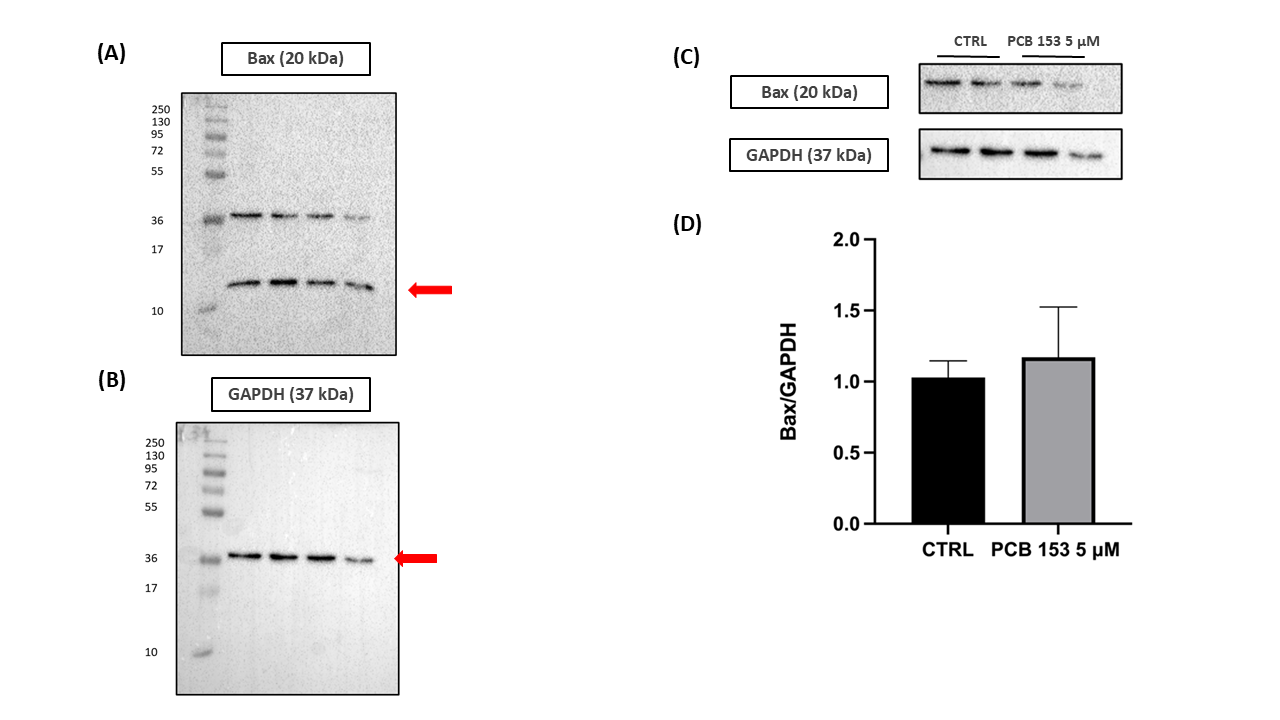

Supplement: Supplementary file 1 [file cells-15-00217-s001.zip › Figure S2 .tif]

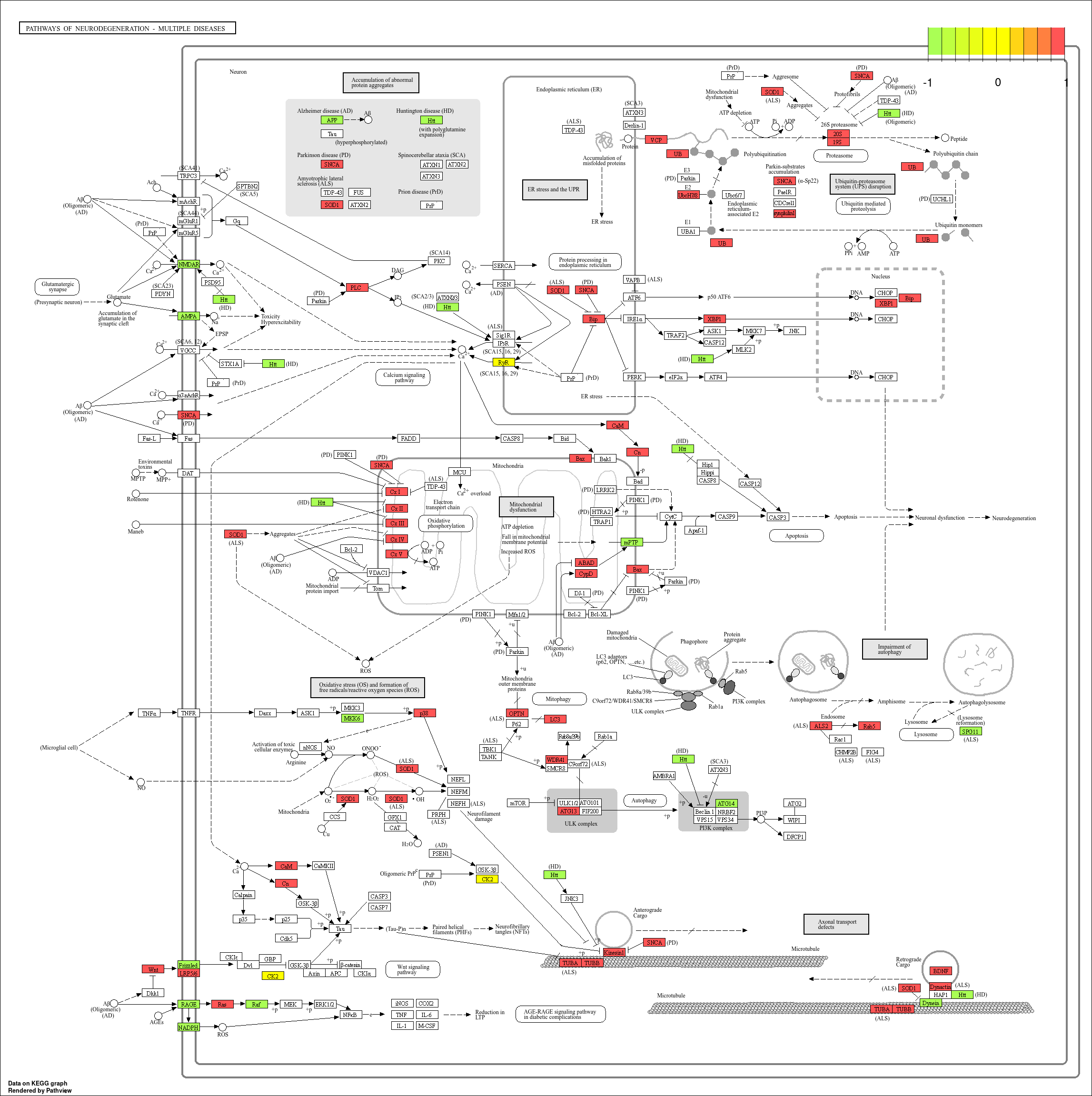

Supplement: Supplementary file 1 [file cells-15-00217-s001.zip › Figure S1.png]

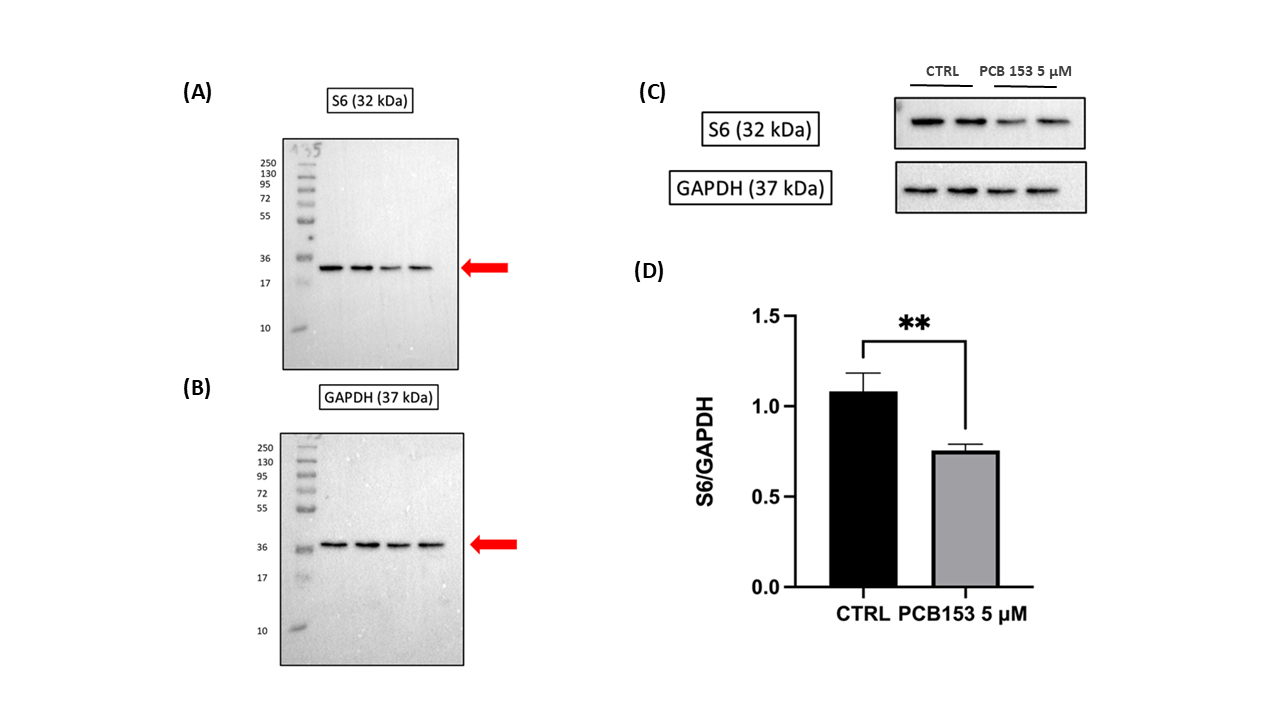

Supplement: Supplementary file 1 [file cells-15-00217-s001.zip › Figure S3.tif]

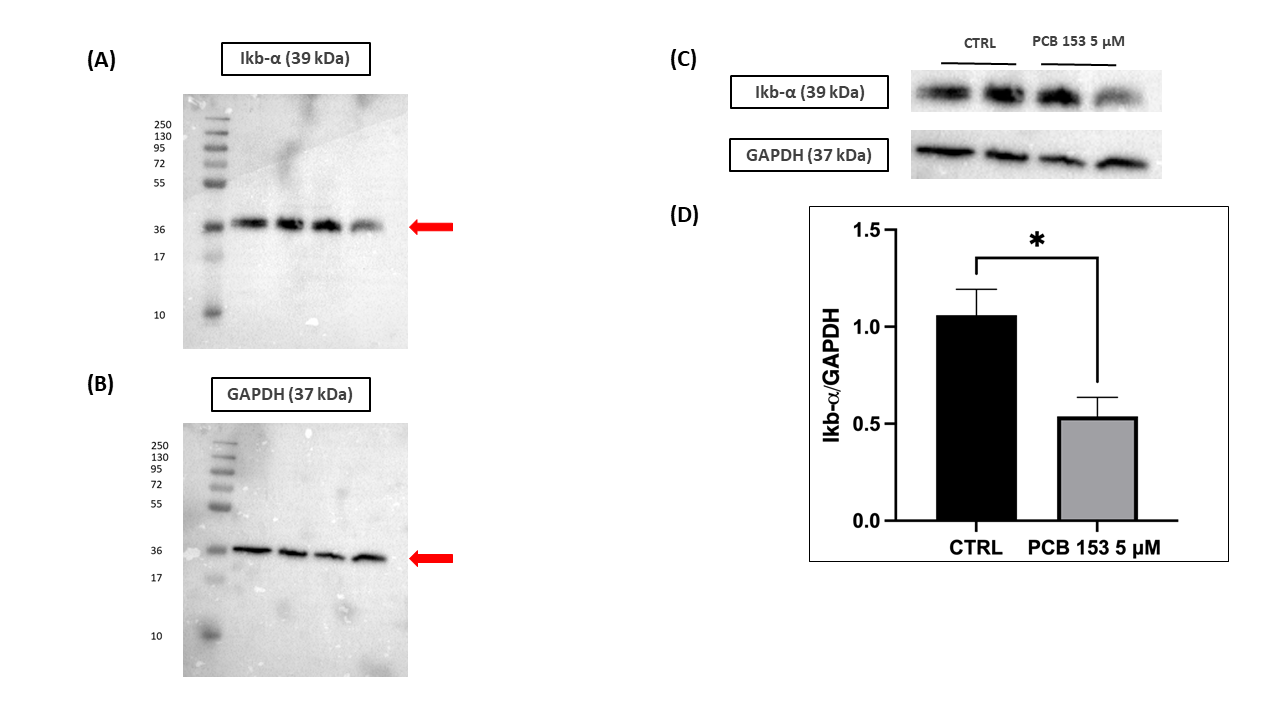

Supplement: Supplementary file 1 [file cells-15-00217-s001.zip › Figure S4.tif]
